# Microsatellite markers for multiple *Pocillopora* genetic lineages offer new insights about coral populations

**DOI:** 10.1038/s41598-017-06776-x

**Published:** 2017-07-27

**Authors:** Yuichi Nakajima, Patricia H. Wepfer, Shohei Suzuki, Yuna Zayasu, Chuya Shinzato, Noriyuki Satoh, Satoshi Mitarai

**Affiliations:** 1 Marine Biophysics Unit, Okinawa Institute of Science and Technology Graduate University, Tancha 1919-1, Onna Okinawa, 904-0495 Japan; 2 Okinawa Marine Science Support Section, Okinawa Institute of Science and Technology Graduate University, Tancha 1919-1, Onna Okinawa, 904-0495 Japan; 3 Marine Genomics Unit, Okinawa Institute of Science and Technology Graduate University, Tancha 1919-1, Onna Okinawa, 904-0495 Japan; 4 Atmosphere and Ocean Research Institute, The University of Tokyo, Kashiwanoha 5-1-5, Kashiwa Chiba, 277-8564 Japan

## Abstract

Population genetics of the coral genus *Pocillopora* have been more intensively studied than those of any other reef-building taxon. However, recent investigations have revealed that the current morphological classification is inadequate to represent genetic lineages. In this study, we isolated and characterized novel microsatellite loci from morphological *Pocillopora meandrina* (Type 1) and *Pocillopora acuta* (Type 5). Furthermore, we characterized previously reported microsatellite loci. A total of 27 loci (13 novel loci) proved useful for population genetic analyses at two sites in the Ryukyu Archipelago, in the northwestern Pacific. Clonal diversity differed in each genetic lineage. Genetic structure suggested by microsatellites corresponded to clusters in a phylogenetic tree constructed from a mitochondrial open reading frame (mtORF). In addition, we found an unknown mitochondrial haplotype of this mtORF. These microsatellite loci will be useful for studies of connectivity and genetic diversity of *Pocillopora* populations, and will also support coral reef conservation.

## Introduction

The genus *Pocillopora*, especially *Pocillopora damicornis*, is the most studied reef-building coral in terms of population genetics^1–9^. This genus includes both broadcast-spawning and brooding species^10^ and it is distributed from the Indian Ocean to the East Pacific. In *Pocillopora*, 17 species are currently recognized in Veron^11^ and in Coral of the World (http://coral.aims.gov.au/), while 22 species are registered in the World Register of Marine Species (WoRMS) (http://www.marinespecies.org/), as of March 2017. However, based on gross morphology, the number of *Pocillopora* species may be overestimated^12^, because recent genetic studies have suggested that the traditional classification may not correspond to genetic species^10, 12–15^. Nevertheless, 16 or more genetic lineages of *Pocillopora* have been considered as putative species based on haplotypes of a mitochondrial DNA region^10, 12, 15^ and genetic structure analyzed using microsatellite markers^10, 15^.

Approximately 400 reef-building coral species inhabit the Ryukyu Archipelago^16^, Japan. A recent phylogenetic approach confirmed six lineages of *Pocillopora* in the northwestern Pacific^12^. *Pocillopora* corals have been damaged by various global and local anthropogenic disturbances, and coral populations have not yet recovered from repeated mass bleaching events since 1998 at reefs in the Ryukyu Archipelago^17^. Population genetics studies using genetic markers make it possible to distinguish invisible inter- and intraspecific relationships among populations. These studies have estimated genetic diversity, differentiation among locations, reproductive characteristics, and species delimitations for various organisms, including corals. Microsatellites, composed of tandemly repeated regions of nucleotides are highly polymorphic among individuals and chromosomes. Next-generation sequencing technology has facilitated development of microsatellite loci, which have recently been developed for reef building corals^18, 19^.

In this study, using next-generation sequencing, we developed cross-lineage microsatellite loci in order to facilitate population genetic analyses for multiple lineages of *Pocillopora* in the Ryukyu Archipelago. Although microsatellites have been already reported in *Pocillopora*, we developed additional markers to widen the choice of markers for population genetics analyses between and within lineages. We developed 13 additional microsatellite markers from morphological *Pocillopora meandrina* and *Pocillopora acuta* colonies. In addition, we validated and characterized microsatellites previously reported from *Pocillopora* for populations in the Ryukyu Archipelago. Those were developed by Magalon *et al*.^20^, Magalon *et al*. unpublished, and Starger *et al*.^21^ and recently, more loci have been isolated by Pinzón & LaJeunesse^13^ and Torda *et al*.^8^. Gorospe & Karl^22^ re-designed primers for two loci, PV7 from Magalon *et al*.^20^ and Pd3-010 from Starger *et al*.^21^ and designated them as Pd2-AB79 and Pd3-EF65, respectively. Using all of these validated microsatellite markers, we genetically characterized populations from multiple lineages of *Pocillopora* from Ueno and Yoshino, Miyako Island, in the Ryukyu Archipelago.

## Results and Discussion

We successfully merged single sequences (2,523,457 reads comprising 1,034,311,164 bp for *P. meandrina* and 7,021,083 reads comprising 2,056,539,596 bp for *P. acuta*) from sequence data derived from genomic DNA. These were used for microsatellite detection and primer design. MISA and Primer3 were employed to identify 1,597 reads with primer sequences, including repeats under the following conditions (4 mer: 10 repeats or more; 5 mer: 8 repeats or more). After removing incomplete or extremely long repeats to facilitate fragment analysis, 752 reads remained (4 mer: 10 to 15 repeats; 5 mer: 8 to 12 repeats). Of these reads, we selected 50 with homologous repeat sequences and flanking regions, from *P. meandrina* and *P. acuta*, to be employed as cross-lineage microsatellite loci. Based on the genetic delimitation and definition by Pinzón *et al*.^10^, these morphological colonies displayed Type 1 and Type 5 mitochondrial open reading frames (mtORF), respectively. The nuclear ribosomal internal transcribed spacer 2 (ITS2) sequence from the Type 1 colony was ITS2 type T (see below).

We confirmed that 13 non-overlapping loci could produce sufficient PCR amplification in all genetic lineages, based on mitochondrial haplotypes derived from fragment analyses (Table 1). In our analyses using previously developed microsatellites, we could not confirm the proper amplification for genotyping of PV3 (Magalon *et al*. unpublished), PV5, PV6^20^, Pd3-002, Pd3-010^21^, or Pd13^8^, which have been widely used in other population genetics studies. However, redesigned primer sequences targeting flanking regions identified in databases, such as Gorospe & Karl^22^ may have improved amplification of the loci, PV7 and Pd3-010. Actually, Pd3-010 was not useful, but Pd3-EF65 by virtue of nested primer sets, was useful even though it targeted the same locus as Pd3-010. Previously reported loci appear to be effective for populations in the Ryukyu Archipelago (Table 2). Clonal replicates were detected at both sites in Type 5 (Table 3). In addition, the population size of Type 5, based on genetic analysis appears to be large in Miyako Island, as in the Yaeyama Islands, where *P. acuta* exists abundantly^23^. Mean values of genetic indices across loci for each lineage/site with large numbers of multilocus genotypes (Types 1, 3, 5 at Ueno, Type 5 at Yoshino) are shown in Table 3. Detailed genetic indices for each locus are in Supplementary Table S1. The *P*_ID_ ranged from 3.3e^–26^ to 1.5e^–20^ (Table 3); therefore, tandem use of these loci allows identification of genets with high resolution. Linkage disequilibrium was not significant (*p* > 0.05) in any microsatellite locus pairs after removal of replicate loci (PV7 is the same locus as Pd2-AB79). Novel microsatellite loci developed here are derived from the nuclear region since it exhibits only moderate heterozygosity and one or two peaks in fragment analysis, excluding scoring errors. Also, polymorphism of zooxanthella loci was confirmed in the same multilocus lineage (MLL). These loci will facilitate combining multiple loci for effective multiplex PCR.

**Table 1:** Characteristics of novel microsatellite loci developed for *Pocillopora* in this study: locus name, repeat motif, forward and reverse primer sequences, and GenBank accession number for each lineage used for isolation of genomic DNA.

Locus	Repeat motif (Type 1/Type 5)	Forward primer sequence (5ʹ–3ʹ)	Reverse primer sequence (5ʹ–3ʹ)	Accession No. (Type 1/Type 5)
Psp_01	(AAGT)2AAGC(AAGT)4N22(TAGA)11/(AAGT)2AAGC(AAGT)9N22(TAGA)10	TCGTTCAATCCACTGACTGC	U19-CCTTTGGATGCGATGTAAT	LC222418/LC222431
Psp_02	(GACCC)9/(GACCC)6	CTGTGCTGGAATTCCCCTTA	U19-AGCCTACGGCGCAATAGTAG	LC222419/LC222432
Psp_10	(TGAG)11(TGGG)3/(TGAG)2TGAA(CGAG)2(TGAG)2(TGGG)4	AGGCGAAGCCATAATGTTGT	U19-CTTCGTTGTGGGCTAAGAGG	LC222420/LC222433
Psp_16	(AAAAC)8/(AAAAC)5	CCCGCTGCTGAGTAAGAATC	U19-AGAGAAACTGCAAAACCGC	LC222421/LC222434
Psp_18	(TGTA)12/(TGTA)9	CACACGTTTTATGACAACGGA	U19-ATAAGCCGTAGGCCCTGTCT	LC222422/LC222435
Psp_23	(TGTA)11/(TGTA)5	ACCATTGCCATCACTGTTCA	U19-TTCATTCATTCGTATTGGCG	LC222423/LC222436
Psp_29	(TTTTC)8/(TTTTC)5	TTTCGTACCAAAATCCAGGC	U19-TTTTTCAGTCGCAAGAGGC	LC222424/LC222437
Psp_32	(CTAT)12/(CTAT)7	AAGCACGCAATTCAGCCTAT	U19-AGCCTAAGACGAATCGAGCA	LC222425/LC222438
Psp_33	(AATC)10/(AATC)6	CCATTTCCCGAATCTCTCTC	U19-CTCGTCGCCCAGATATAAA	LC222426/LC222439
Psp_35	(CTAT)3CTAC(CTAT)10TTAT(CTAT)3/(CTAT)16	TGGCTGATGTCTGTGGGTAA	U19-CGCGATTATCGAAAGTTTG	LC222427/LC222440
Psp_39	(AAGTG)8/(AAGTG)4	TCTTTACAGCACAGGAGCCA	U19-TTTTTCTTGCGGTCCAATTC	LC222428/LC222441
Psp_41	(ATTT)10/(ATTT)3ACTT(ATTT)3	U19-CGCACAAGGAAAATTTGTT	TTCCACACCAGAAGATGACG	LC222429/LC222442
Psp_48	(CTTT)11/(CTTT)4	TGTAAATTCAAGAGAATGGGCA	U19-GTTTCCTGATGGTGTTCT	LC222430/LC222443

**Table 2:** Characteristics of previously developed microsatellite loci for *Pocillopora*: locus name, forward and reverse primer sequences, and GenBank accession number.

Locus	Forward primer sequence (5ʹ-3ʹ)	Reverse primer sequence (5ʹ-3ʹ)	Accession No.	Reference
Pd2-001	U19-CAGACTTGTCGGAATGAAAGC	TTTTGTTTATAAGTCGATACAATGCA	DQ684672	Starger *et al*.^21^
Pd3-002*	U19-ATCCGAATACAAGCGAAACG	CAAAGCTTCTATCAGAAAATGCAA	DQ684673	Starger *et al*.^21^
Pd2-003*	U19-CCTCTTCCTGTTTGGGCTCT	TCTGCATTACGTTTGTTTGACA	DQ684674	Starger *et al*.^21^
Pd3-004	U19-ACCAGACAGAAACACGCACA	GCAATGTGTAACAGAGGTGGAA	DQ684675	Starger *et al*.^21^
Pd3-005	U19-AGAGTGTGGACAGCGAGGAT	GTTCCTTCGCCTTCGATTTT	DQ684676	Starger *et al*.^21^
Pd2-006	U19-ATCTCCATGTGATCGGCATT	GTTCCCCCAGCTGAGAAGTT	DQ684677	Starger *et al*.^21^
Pd2-007	U19-AAGAAGGTGTGGTATTTCAGAGGG	GGTGGATAAAGTATTTCTCACTCTTGG	EF120462	Starger *et al*.^21^
Pd3-008	AGTTGAGGTTGTTGAAACATG	U19-TCCATGCAGAACCCC	EF120463	Starger *et al*.^21^
Pd3-009	U19-CCAATGCGTCCGTAGCTCTC	ATCACCTAAAAATTTCAGTCCCTTACC	EF120464	Starger *et al*.^21^
Pd3-010*	U19-CTGATCAACAAACTGGGAGGC	TCATTAGAAATCATCTTGATTTGATAAGG	EF120465	Starger *et al*.^21^
PV2	U19-CCAGGACCCATTTATACTCC	TGCAGTGTTCTACTTGTCAGTGC	AY397777	Magalon *et al*.^20^
PV3*	U19-TGAAACAGGATTGACGACGA	AACCCGAATGATTCCACAAT	AY397778	Magalon *et al*. (unpublished)
PV5*	U19-GTCATCACGCAAAGTTCC	GAATAGCCTGCGTTTATTTGG	AY397780	Magalon *et al*.^20^
PV6*	U19-CTTTCCCGACCAGTTTAGGG	AGCCGTTCAGCTACCTATGG	AY397781	Magalon *et al*.^20^
PV7	U19-GAGATGGATGGAGACTGC	GGTATCTCTGTGCTCAGTTCTTTG	AY397782	Magalon *et al*.^20^
Pd2-AB79 (from PV7)	GGAGATGGATGGAGACTGCT	U19-AGTGCACGCACTAGATAGA	AB214379	Gorospe & Karl^22^
Pd3-EF65 (from Pd3-010)	U19-TGTGCAGGTGTTGTGACTGA	TGTCTTTTTCACTTTTGCTTCAA	EF120465	Gorospe & Karl^22^
Pd4	U19-ACGCACACAAACCAACAAAC	TAATTCCATCAACTCAAAGGGG	Not found	Torda *et al*.^8^
Pd11	TCGTTTGAAGGGAAATGCTC	U19-GCATGCTATGTATGCGAGA	Not found	Torda *et al*.^8^
Pd13	TGTTCCTCTCTTTCTCTCTTCCA	U19-CATTTATGTTCCTTTCACGGC	Not found	Torda *et al*.^8^
Poc40	U19-TTATTATATGGGTGTATGC	CTCAAAGTGCGATTAAAGCC	Not found	Pinzón & Lajeunesse^13^

*We could not confirm the proper amplification for genotyping.

**Table 3:** The number of samples (*N*), the number of multilocus genotypes (*G*), the number of multilocus lineages (*N*_MLL_), the mean number of alleles (*N*_A_), observed (*H*_O_) and expected (*H*_E_) heterozygosities, and the deviation index from Hardy-Weinberg equilibrium (*F*_IS_) across 27 loci for each lineage or site. The probability of identity (*P*_ID_) for 27 loci was determined with GenAlEx.

Site	Lineage	*N*	*G*	*N*_MLL_	*N*_A_	*H*_O_	*H*_E_	*F*_IS_	*P*_ID_
Ueno	Type 3	1	1	1	—	—	—	—	—
	Type 5	29	16	10	4.48	0.524	0.584	0.103	1.5e^−20^
Yoshino	Type 1 (ITS2 type T)	11	11	11	6.07	0.530	0.595	0.107	3.3e^−23^
	Type 1 (ITS2 type C)	11	11	11	5.85	0.519	0.615	0.170	1.8e^−23^
	Type 3	20	20	20	7.82	0.539	0.648	0.172	3.3e^−26^
	Type 4	1	1	1	—	—	—	—	—
	Type 5	10	8	8	4.67	0.526	0.596	0.106	3.0e^−21^
	Type 8	1	1	1	—	—	—	—	—
	Unknown	6	5	3	—	—	—	—	—

For lineages or sites with low numbers of MLLs, *N*_A_, *H*_O_, *H*_E_, *F*_IS_, and *P*_ID_ were not calculated in this table. Values of *N*_A_, *H*_O_, *H*_E_, and *F*_IS_ for each locus are shown in Supplementary Table S1. PV7 was removed from this analysis because it was identical to Pd2-AB79.

**Figure 1:**
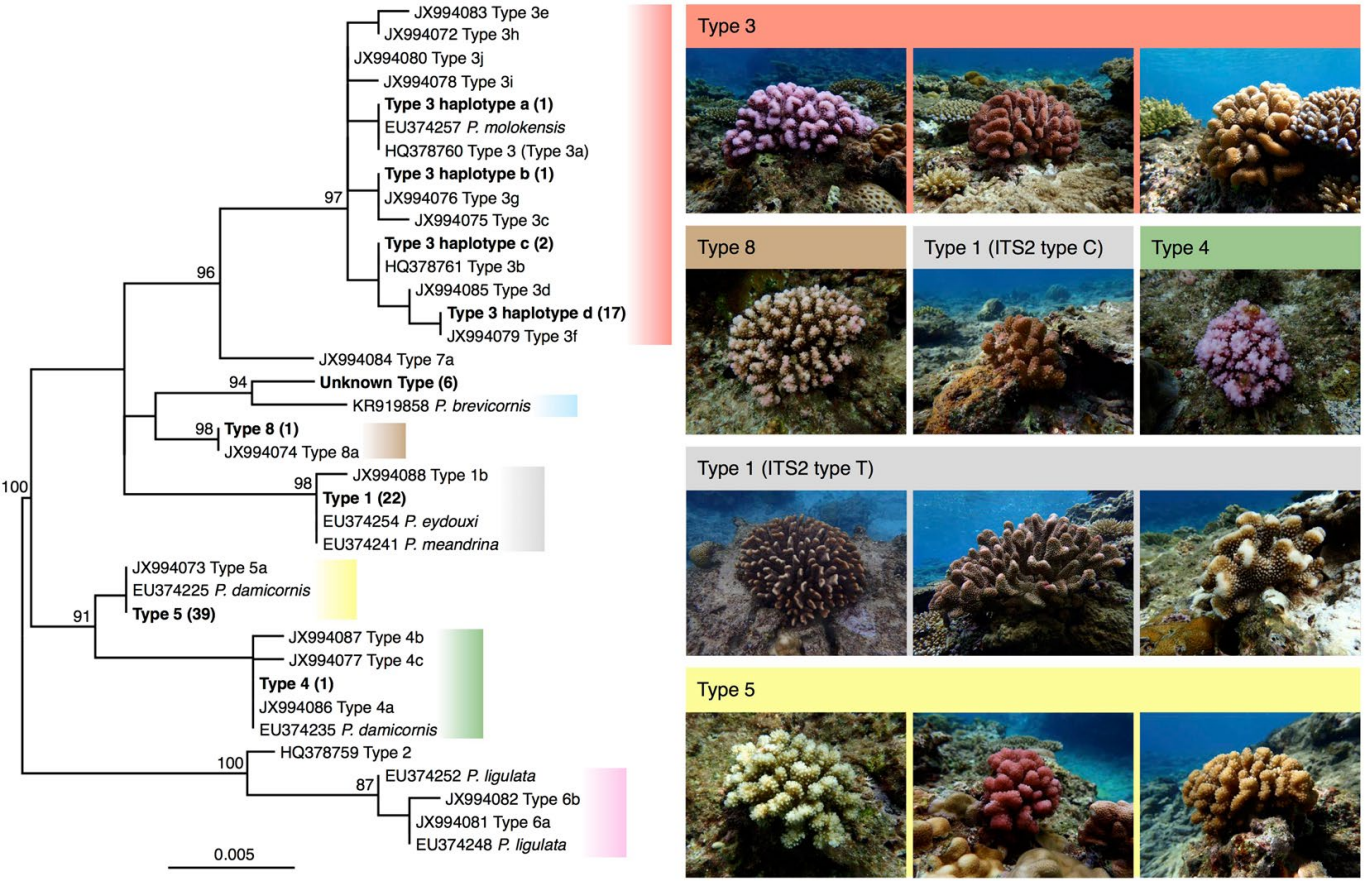
Maximum likelihood phylogenetic tree based on the mtORF comparing the variety of haplotypes with the variety of morphological types of *Pocillopora*. Immense diversity is evident even within lineages in the Ryukyu Archipelago. Boldface indicates haplotypes that were isolated during this study (GenBank accession No. LC222444–LC222452). Values in parentheses indicate the number of colonies collected from two sites at Miyako Island. Values in the tree indicate maximum likelihood bootstrapping (over 75%). Cluster colors correspond to genetic lineages reported by Schmidt-Roach *et al*.^12^.

Mitochondrial data showed two haplotype groups (Types 3 and 5) at the Ueno site, and haplotype groups of mtORF (Types 1, 3, 4, 5, 8, and unknown) at the Yoshino site. Furthermore, Type 1 was separated into two haplotype groups based upon ITS2. At least six lineages of *Pocillopora* inhabit the northwestern Pacific^12^. This study confirmed that all of these, plus one more, occur in the Ryukyu Archipelago. One unknown haplotype sequence was related to a sequence from *Pocillopora brevicornis* (GenBank accession No. KR919858, Mayfield *et al*. unpublished; see also Schmidt-Roach *et al*.^12^ about *Pocillopora* cf. *brevicornis*), which differed by 5 bp. A maximum likelihood phylogenetic tree based on mtORF haplotypes was constructed (Fig. 1).

**Figure 2:**
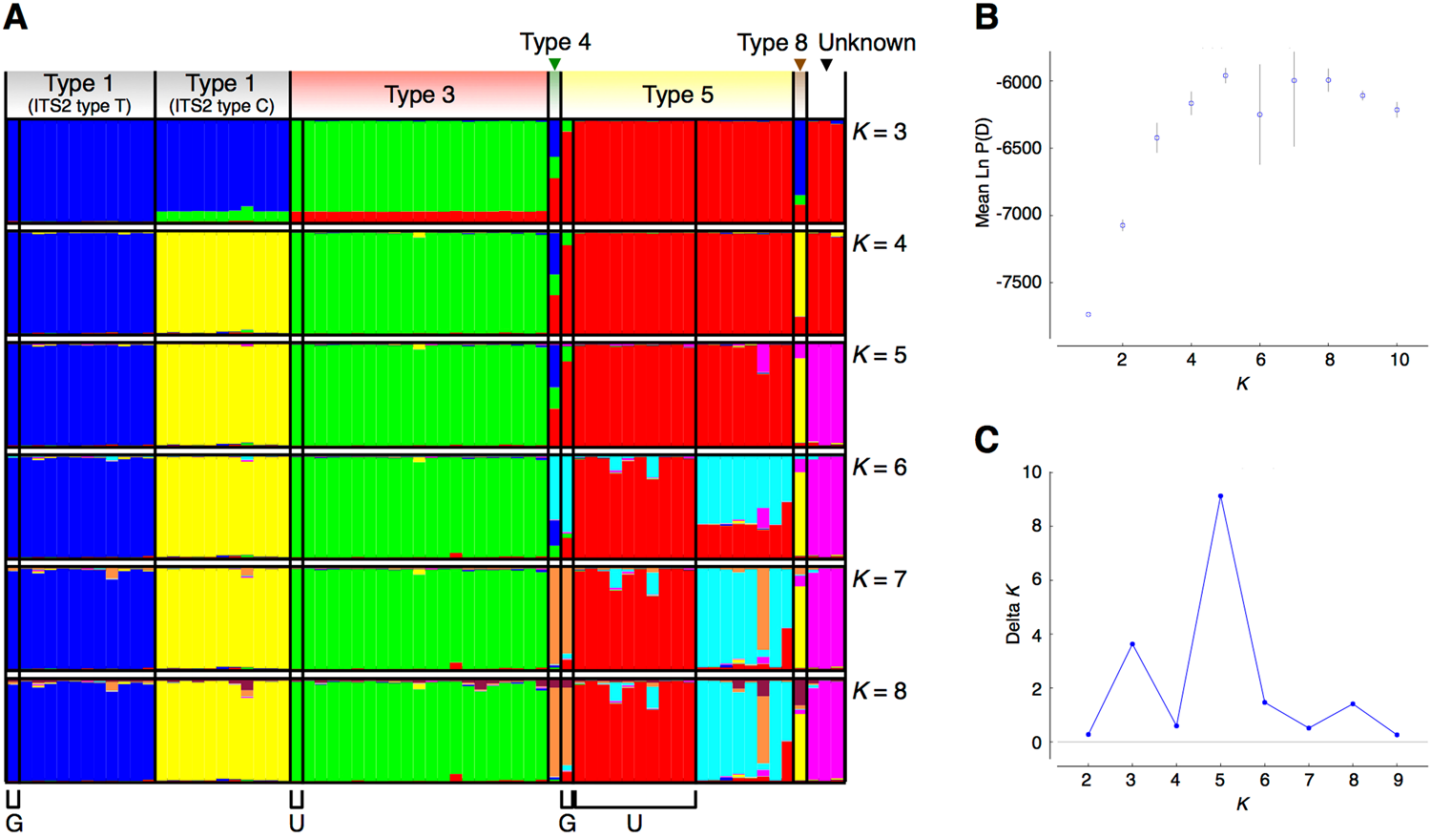
Population genetic assessment using STRUCTURE suggests five probable genetic clusters within just the pair of sites we surveyed. These clusters reflect primarily the mtORF (including ITS2) types. The Evanno method was used to decide the probable number of genetic clusters. (**A**) Each bar plot shows the possible membership of genetic clusters for each MLL. Those ranged from three to eight (*K* = 3 to *K* = 8), with *K* = 5 being optimal. G: MLLs of samples collected from the western coast of Okinawa Island, used for Illumina sequencing, U: MLLs of samples collected at Ueno, Miyako Island. Samples of other MLLs were collected at Yoshino, Miyako Island. (**B** and **C**) The model criterion of choice to detect the most probable number of genetic clusters (*K* = 1 to *K* = 10) across 10 iterations per *K* (the assumed number of clusters). Graphs of (**B**) mean log probability (Ln P(D)) and (**C**) Δ*K* values based on the rate of change in Ln P (**D**) between successive *K* values (*K* = 2 to *K* = 9) for detecting the most probable number of *K*.

Genetic structure among genetic lineages separated on the basis of mtORF and based on 27 microsatellite loci (Fig. 2), supported the results of Pinzón *et al*.^10^. Using the method of Evanno *et al*.^24^ to determine the most probable number of genetic populations (*K*), the largest peak of Δ*K* was *K* = 3 (Fig. 2). Otherwise, Δ*K* values at *K* = 5 and *K* = 8 also showed small peaks; therefore, we showed genetic population structure for values of *K* ranging from 3 to 8 (Fig. 2). Genetic structure among Type 1 mtORFs was reflected in different ITS2 haplotypes, as reported by Pinzón *et al*.^10^. We defined ITS2 types T and C within mtORF Type 1, based on their haplotype groups (see registered sequences: Genbank accession No. LC222453– LC222460). Some MLLs in mtORF Type 5 were assigned to the cluster containing Type 4. However, there was only one mtORF Type 4 sample. Further studies with additional samples are needed to fully distinguish these two lineages. Also, when *K* ≥ 6 in STRUCTURE analysis, definite genetic structure was detected, even between sites within mtORF Type 5, despite the close geographic proximity of these populations, ~17 km along the coast. Thus, genetic structure even in the same genetic lineage at fine geographic scale could be confirmed in a brooding species^25^. In part, genetic differentiation within a given lineage may reflect differences of water depth (<~17 m) between sites.

In conjunction with primers developed previously, these new primer pairs also may be used for a variety of *Pocillopora* lineages (including various morphological species) and may provide useful information about coral population genetics in general. By sampling a greater number of specimens across a larger geographic area, these microsatellite loci will be beneficial for population genetics within and between *Pocillopora* lineages in various localities. They will be helpful to understand genetic structure and population dynamics at contemporary and historical time scales, and for parentage analysis, identification of evolutionary lineages, and genetic diversity to understand maintenance of coral populations.

This study suggested that 13 new and 14 previously reported loci are useful as cross-lineage microsatellites for population genetic analyses of *Pocillopora* in the Ryukyu Archipelago. mtORF Type 5 obtained at two sites for validation of microsatellites indicated large number of clonal replicates, especially in Ueno. We detected distinct genetic structure among lineages, as estimated from mtORF haplotypes (ITS2 was also considered for Type 1). Although Schmidt-Roach *et al*.^12^ reported that at least six lineages of *Pocillopora* inhabit the northwestern Pacific region, this study indicated that all six occur in the Ryukyu Archipelago, despite sampling only two sites. We confirmed one more lineage with an unknown haplotype in this region. Additional genetic diversity may be discovered in this region by extending the number and geographical distribution of sampling sites. More extensive analyses of genetic diversity and connectivity in within and between populations will be needed to better understand population maintenance and dynamics for conservation and management of coral reef organisms.

## Methods

### Next-generation sequencing and isolation of microsatellites for two *Pocillopora* lineages

We collected branches from the morphological species, *P. meandrina* and *P. acuta*, along the western coast of Okinawa Island in the Ryukyu Archipelago, a subtropical area located in the northwestern Pacific Ocean, Japan. These belonged to mitochondrial open reading frame (mtORF) Type 1 and Type 5 (see Results and Discussion). Specimens were preserved in ethanol and genomic DNA was extracted using a DNeasy Blood & Tissue Kit (Qiagen) following the standard protocol. We sequenced 250-bp paired-end reads using a MiSeq sequencer (Illumina) according to the manufacturer’s instructions. After merging single-end sequences using PEAR ver. 0.9.8^26^, to isolate reads of 100 bp or more having repeat sequences of *P. meandrina* (Type 1), we used MISA (http://pgrc.ipk-gatersleben.de/misa/) to detect simple sequence repeats and to design PCR primers using Primer3 (http://pgrc.ipk-gatersleben.de/misa/primer3.html). We searched *P. acuta* (Type 5) using BLAST+ ver. 2.3.0^27^. We confirmed that sequence regions, including microsatellites, were conserved between the two lineages.

### Collection of coral samples, sequencing of mtORF, and phylogenetic analysis of *Pocillopora*

To confirm validation and polymorphism of microsatellite loci, branches from 90 *Pocillopora* colonies were collected at Ueno (30 colonies; 24°43ʹ09″ N, 125°20ʹ27″ E; <1 m depth, by walking along the seashore) and Yoshino (60 colonies; 24°44ʹ55″ N, 125°26ʹ45″ E; <18 m depth, by SCUBA diving) at Miyako Island at the Ryukyu Archipelago, Japan. Genomic DNA was extracted from preserved coral branches as described above. Mitochondrial haplotypes were confirmed by sequencing the mtORF region^28^. PCR reaction mixtures consisted of: (10 μL) containing <30 ng/μL template DNA, AmpliTaq Gold 360 Master Mix (Thermo Fisher Scientific), and the primers (final concentration: 2 μM for each primer) for mtORF: FATP6.1 (5ʹ-TTTGGGSATTCGTTTAGCAG-3ʹ) and RORF (5ʹ-SCCAATATGTTAAACASCATGTCA-3ʹ)^29^. The PCR protocol consisted of 94 °C for 1 min, followed by 40 cycles at 94 °C for 30 s, 53 °C for 30 s, and 72 °C for 75 s, with a final extension at 72 °C for 5 min. After reaction of Exonuclease I (Takara) and Shrimp Alkaline Phosphatase (Takara) to clean up PCR products, sequencing was conducted by Macrogen Japan.

mtORF sequences obtained were aligned with others from previous studies^10, 13, 28, 29^ including unpublished work of Mayfield *et al*., using MUSCLE in MEGA ver. 6.06^30^. The molecular evolution model was selected by MEGA, and the best model was determined to be the HKY (Hasegawa-Kishino-Yano) model with a gamma distribution of rate variation across sites (+G). Rapid bootstrap analysis employing the maximum likelihood method for phylogenetic analysis was carried out using MEGA with the model using 1000 bootstrap replicates. The phylogenetic tree was drawn in FigTree ver. 1.4.2 (http://tree.bio.ed.ac.uk/software/figtree/).

The nuclear ribosomal internal transcribed spacer 2 (ITS2) was used for identification of less differentiated genetic lineages within Type 1^10, 29^. PCR reaction mixtures comprised: (10 μL) containing <30 ng/μL template DNA, AmpliTaq Gold 360 Master Mix, and primers (final concentration: 2 μM for each primer) of ITS2: ITSc2-5 (5ʹ-AGCCAGCTGCGATAAGTAGTG-3ʹ) and R28S1 (5ʹ-GCTGCAATCCCAAACAACCC-3ʹ)^29^. Conditions for PCR and sequencing were the same as for mtORF.

### Genotyping of *Pocillopora* using novel and known microsatellite loci

Together with novel microsatellite loci developed here, we characterized loci for *Pocillopora* previously developed by Magalon *et al*.^20^, Magalon *et al*. (unpublished), Starger *et al*.^21^, Pinzón & LaJeunesse^13^, Torda *et al*.^8^, and two loci modified by Gorospe & Karl^22^. For scoring of microsatellite genotypes of each colony, the PCR reaction mixture (5 μL) contained template DNA (<30 ng/μL), AmpliTaq Gold 360 Master Mix, and three primers for each locus: a non-tailed reverse primer (0.5 μM), a forward primer with a sequence tail of U19 (5ʹ-GGTTTTCCCAGTCACGACG-3ʹ) (0.5 μM), and a U19 primer (0.5 μM) fluorescently labeled with FAM, VIC, NED, or PET^31^. The PCR protocol consisted of 95 °C for 9 min, followed by 35 cycles at 95 °C for 30 s, 56 °C for 30 s, and 72 °C for 1 min, with a final extension at 72 °C for 5 min. We furthermore conducted PCR using microsatellites for zooxanthellae, C1.02^32^, SymC_3-02, SymC_3-03^33^, and D1Sym11^34^ within the same MLL to confirm that the microsatellites were derived from nuclear loci, since different symbiont genotypes have been detected from the same MLL in *Pocillopora* ^35^. PCR conditions were the same as for microsatellites from colonies, except for the annealing temperature (50 °C for 4 zooxanthellae microsatellites). PCR products were analyzed using an ABI 3730 capillary DNA sequencer (Thermo Fisher Scientific) with the GeneScan 600 LIZ size standard (Thermo Fisher Scientific) to identify genotypes by the length of the amplicon. Fragment size and intensity were confirmed using Geneious ver. 9.0.4 (Biomatters).

### Data analyses to characterize microsatellites and populations

The concept of multilocus lineage (MLL) was employed to avoid underestimating clonality due to genotyping errors and somatic mutations^36^. If genotypes of ≤3 loci differed and all other loci coincided, a colony was considered to be clonal, derived by asexual reproduction. Either a scoring error or a somatic mutation was assumed to be responsible for the variable locus/loci. The number of different loci was calculated using GenAlEx ver. 6.501^37^. When different genotypes were determined to belong to the same MLL, the most common genotype was assigned^38^. However, if the most common genotype could not be determined (*e.g*., if two genotypes occurred in equal numbers), the genotype was set to zero^39^. We also calculated *P*_SEX_ to estimate the possibility of coincidence of multilocus genotypes by sexual reproduction, using GenClone ver. 2.0^40^. After removing clonal replicates from the data set, the number of alleles, values of observed and expected heterozygosity (*H*_O_ and *H*_E_, respectively), and deviation index (*F*_IS_) from Hardy-Weinberg equilibrium were evaluated for each locus and lineage using GenAlEx. Lineages with few MLLs were not used for calculation of these genetic indices. The probability of identity (*P*_ID_) was also calculated using GenAlEx to estimate the resolution of loci for each lineage/site. Linkage disequilibrium was estimated for each lineage using Genepop on the Web^41, 42^.

Genetic structure based on Bayesian clustering was inferred using STRUCTURE ver. 2.3.4^43^. A burn-in period of 100,000 followed by 1,000,000 Markov chain Monte Carlo (MCMC) replications was used for population clustering without LOCPRIOR (prior sampling location information) model under the admixture model and assuming independent allele frequencies^44^. After calculation of the mean log probability, Ln P(D), to estimate the most probable number of genetic clusters, the number of clusters was determined using the method of Evanno *et al*.^24^, as implemented in STRUCTURE HARVESTER^45^. Run data were merged using CLUMPAK^46^.

## Electronic supplementary material

Supplementary Table S1
