# Supplementary material for: Microsatellite markers for multiple Pocillopora genetic lineages offer new insights about coral populations
Source: Sci Rep. 2017 Jul 27;7:6729. doi: 10.1038/s41598-017-06776-x (PMC5532301; doi:10.1038/s41598-017-06776-x)
Supplement: Supplementary file 1 — Supplementary Table S1 [file 41598_2017_6776_MOESM1_ESM.pdf]

Microsatellite markers for multiple *Pocillopora* genetic lineages offer new insights about coral populations

Yuichi Nakajima [1,*], Patricia H. Wepfer [1], Shohei Suzuki [2], Yuna Zayasu [3], Chuya Shinzato [3,4], Noriyuki Satoh [3], Satoshi Mitarai [1]

1 Marine Biophysics Unit, Okinawa Institute of Science and Technology Graduate University, Tancha 1919-1, Onna, Okinawa 904-0495, Japan

2 Okinawa Marine Science Support Section, Okinawa Institute of Science and Technology Graduate University, Tancha 1919-1, Onna, Okinawa 904-0495, Japan

3 Marine Genomics Unit, Okinawa Institute of Science and Technology Graduate University, Tancha 1919-1, Onna, Okinawa 904-0495, Japan

4 Atmosphere and Ocean Research Institute, The University of Tokyo, Kashiwanoha 5-1-5, Kashiwa, Chiba 277-8564, Japan

*Corresponding author

Phone: +81-98-966-1669

e-mail: yuichi.nakajima@oist.jp; yuichi.nakajima@outlook.com

Supplementary Table S1. Allele size range of amplification products, including the U19 sequence (range). The number of samples after removal of clonemates accounts for multilocus lineages (*N* for MLL), the number of alleles (N_A), observed (H_O) and expected (H_E) heterozygosities, and deviation index from Hardy-Weinberg equilibrium (F_{IS}) for each locus/lineage/site. Cell color of pale gray: $p < 0.05$, gray: $p < 0.01$, black: $p < 0.001$.

Microsatellites from this study

**Lineage/site**		**Psp_01**	**Psp_02**	**Psp_10**	**Psp_16**	**Psp_18**	**Psp_23**	**Psp_29**	**Psp_32**	**Psp_33**	**Psp_35**	**Psp_39**	**Psp_41**	**Psp_48**
Type 1 (ITS2 type T)	Range	223–296	241–292	97–141	172–193	261–309	160–200	252–267	127–208	259–284	216–268	124–159	247–283	185–208
Yoshino	*N* for MLL	11	11	11	11	11	11	11	11	11	11	11	11	11
	N_A	13	11	5	8	7	6	4	10	8	10	4	9	6
	H_O	0.545	0.909	0.727	0.273	0.909	0.727	0.636	0.636	0.818	0.818	0.364	0.636	0.818
	H_E	0.913	0.860	0.587	0.789	0.798	0.744	0.657	0.872	0.773	0.806	0.492	0.814	0.756
	F_{IS}	0.403	–0.058	–0.239	0.654	–0.14	0.022	0.031	0.270	–0.059	–0.015	0.261	0.218	–0.082
Type 1 (ITS2 type C)	Range	211–240	241–246	97–125	167–188	285–341	160–184	241–262	127–253	263–289	212–292	124–134	243–259	181–228
Yoshino	*N* for MLL	9	11	11	11	11	11	11	10	11	11	11	10	8
	N_A	7	2	5	6	11	3	5	12	9	12	2	4	4
	H_O	0.333	0.182	0.455	0.636	0.909	0.364	0.636	0.700	0.636	1.000	0.000	0.300	0.125
	H_E	0.747	0.165	0.632	0.773	0.888	0.310	0.702	0.895	0.835	0.872	0.298	0.685	0.555
	F_{IS}	0.554	–0.1	0.281	0.176	–0.023	–0.173	0.094	0.218	0.238	–0.147	1.000	0.562	0.775
Type 3	Range	215–333	251–302	113–129	173–189	265–368	152–184	252–262	123–260	263–292	196–276	119–124	251–285	184–240
Yoshino	*N* for MLL	19	20	20	20	19	20	20	20	15	20	20	20	19
	N_A	15	11	4	10	19	8	3	18	9	2	3	9	14
	H_O	0.368	0.950	0.500	0.850	0.842	0.800	0.250	0.900	0.667	0.050	0.050	0.500	0.895
	H_E	0.914	0.869	0.629	0.856	0.925	0.709	0.226	0.918	0.540	0.049	0.636	0.784	0.834
	F_{IS}	0.597	–0.094	0.205	0.007	0.090	–0.129	–0.105	0.019	–0.235	–0.026	0.921	0.362	–0.073
Type 5	Range	227–272	236–287	113	173–188	265–350	152–160	243–271	127–160	259–267	216–268	124	251–271	185
Ueno	*N* for MLL	9	9	10	10	7	10	10	10	10	10	10	10	10
	N_A	3	5	1	6	9	4	4	8	3	8	1	5	1
	H_O	0.778	0.556	0.000	0.700	1.000	0.600	0.600	1.000	0.400	0.500	0.000	0.300	0.000
	H_E	0.537	0.704	0.000	0.650	0.847	0.625	0.695	0.850	0.540	0.805	0.000	0.675	0.000
	F_{IS}	–0.448	0.211	–	–0.077	–0.181	0.040	0.137	–0.176	0.259	0.379	–	0.556	–
Type 5	Range	227–280	236	113–133	175–190	269–341	152–173	243–262	123–143	257–271	216–276	124	251–259	181–251
Yoshino	*N* for MLL	8	8	8	8	7	8	8	8	8	8	8	8	8
	N_A	7	1	2	5	8	6	3	5	4	10	1	3	4
	H_O	0.750	0.000	0.250	0.875	0.571	0.625	0.250	0.625	0.375	0.625	0.000	0.250	0.375
	H_E	0.766	0.000	0.219	0.742	0.827	0.688	0.617	0.563	0.672	0.883	0.000	0.227	0.492
	F_{IS}	0.020	–	–0.143	–0.179	0.309	0.091	0.595	–0.111	0.442	0.292	–	–0.103	0.238

Microsatellites from previous studies

**Lineage/site**		**Pd2–001**	**Pd3–004**	**Pd3–005**	**Pd2–006**	**Pd2–007**	**Pd3–008**	**Pd3–009**	**PV2**	**PV7**	**Pd2–AB79**	**Pd3–EF65**	**Pd4**	**Pd11**	**Pd13**	**Poc40**
Type 1 (ITS2 type T)	Range	213–223	181–190	211–232	207–219	276–440	169–172	347–356	146–147	207–262	158–178	201–225	155–160	144–159	166–170	309–321
Yoshino	*N* for MLL	11	11	11	11	11	11	10	10	11	11	11	11	11	5	11
	N_A	5	3	5	6	7	2	2	2	9	8	9	3	6	2	3
	H_O	0.545	0.182	0.364	0.455	0.818	0.273	0.100	0.000	0.545	0.727	0.909	0.091	0.545	0.200	0.273
	H_E	0.496	0.169	0.388	0.508	0.789	0.236	0.095	0.180	0.835	0.835	0.839	0.541	0.711	0.180	0.244
	F_{IS}	–0.1	–0.073	0.064	0.106	–0.037	–0.158	–0.053	1.000	0.347	0.129	–0.084	0.832	0.233	–0.111	–0.119
Type 1 (ITS2 type C)	Range	213–225	172–193	239–257	209–211	252–553	172–175	347–356	138–180	201–248	156–168	201–222	152–161	146–159	162–193	315–323
Yoshino	*N* for MLL	11	11	11	11	11	11	11	11	10	11	11	11	11	11	11
	N_A	7	8	7	2	11	2	3	3	6	5	7	4	7	6	4
	H_O	0.727	0.909	0.818	0.545	0.818	0.091	0.545	0.091	0.600	0.455	0.727	0.364	0.636	0.364	0.636
	H_E	0.690	0.793	0.798	0.463	0.876	0.087	0.475	0.244	0.600	0.550	0.785	0.707	0.682	0.455	0.657
	F_{IS}	–0.054	–0.146	–0.026	–0.179	0.066	–0.048	–0.148	0.627	0.000	0.173	0.074	0.485	0.067	0.200	0.031
Type 3	Range	211–221	178–190	214–242	205–217	276–391	169–181	347–362	146–172	244–256	160–172	195–219	148–161	165–188	162–174	312–329
Yoshino	*N* for MLL	20	20	20	20	20	20	20	16	20	20	20	19	20	20	20
	N_A	7	3	8	7	7	5	5	4	6	6	9	5	10	6	4
	H_O	0.600	0.050	0.750	0.450	0.350	0.350	0.600	0.375	0.550	0.700	0.900	0.368	0.600	0.400	0.450
	H_E	0.716	0.226	0.734	0.519	0.521	0.529	0.619	0.604	0.786	0.783	0.853	0.724	0.854	0.485	0.431
	F_{IS}	0.162	0.779	–0.022	0.133	0.329	0.338	0.030	0.379	0.300	0.105	–0.056	0.491	0.297	0.175	–0.043
Type 5	Range	213–215	172–181	223–248	207–213	244–556	172–178	344–368	146–199	244–246	160–162	201–219	149–161	169–171	158–193	309–335
Ueno	*N* for MLL	10	10	10	10	10	10	10	10	10	10	10	10	10	10	9
	N_A	2	4	7	4	6	3	6	4	2	2	6	3	3	6	7
	H_O	0.200	0.600	0.900	0.100	1.000	0.600	0.700	0.600	0.300	0.300	0.900	0.600	0.100	0.900	0.222
	H_E	0.420	0.510	0.775	0.665	0.755	0.585	0.745	0.615	0.255	0.255	0.760	0.580	0.605	0.770	0.796
	F_{IS}	0.524	–0.176	–0.161	0.850	–0.325	–0.026	0.060	0.024	–0.176	–0.176	–0.184	–0.034	0.835	–0.169	0.721
Type 5	Range	213–219	172–178	220–239	209–215	253–487	172–181	344–356	146–207	244–254	160–170	207–222	156–161	150–173	158–208	309–335
Yoshino	*N* for MLL	8	8	8	8	8	8	8	8	8	8	8	8	8	8	8
	N_A	4	3	6	4	9	4	4	6	4	4	5	2	6	5	5
	H_O	0.375	0.625	0.875	0.500	0.750	0.500	0.625	0.750	0.750	0.750	0.875	0.250	0.750	0.750	0.250
	H_E	0.695	0.539	0.734	0.688	0.719	0.695	0.648	0.688	0.633	0.633	0.727	0.375	0.781	0.742	0.742
	F_{IS}	0.461	–0.159	–0.191	0.273	–0.043	0.281	0.036	–0.091	–0.185	–0.185	–0.204	0.333	0.040	–0.011	0.663
